# Target drug delivery system as a new scarring modulation after glaucoma filtration surgery

**DOI:** 10.1186/1746-1596-6-64

**Published:** 2011-07-08

**Authors:** Tingting Shao, Xiaoning Li, Jian Ge

**Affiliations:** 1 Department of Ophthalmology and Vision Science, Eye and Ear Nose Throat Hospital, Shanghai Medical School, Fudan University, No. 83, Fenyang Road, Shanghai, PR China; 2 State Key Laboratory of Ophthalmology, Zhongshan Ophthalmic Center, Sun Yat-sen University, 54 Xian Lie Nan Road, 510060 Guangzhou, Guangdong, PR China

## Abstract

**Background:**

Excessive wound healing following glaucoma filtration surgery is the main determinant of surgical failure, resulting from the activation of human Tenon's capsule fibroblasts (HTFs). To mitigate the excessive wound healing, the topicall use of antiproliferative agents, such as mitomycin C (MMC) and 5-fluorouracil (5-FU), has increased the surgery success rate, but the traditional administration of these agents can result in a variety of toxicities with nonspecific damage. However, modulation of the wound healing process to prevent excessive fibroblast proliferation and scar formation can play a major role in improving the outcome of surgery. Therefore, the search for alternative modes of drug delivery and new agents is needed to minimize the ocular complications and improve the success of surgery. We have shown that there is a postoperative overexpression of the LDL receptor (LDLr) in the activated HTFs may provide a novel target for drug delivery systems.

**Presentation of the Hypothesis:**

We hypothesize that antifibrotic agents (MMC) encapsulated in LDLr targeting drug delivery system (LDL-MMC-chitosan nanoparticles) may be proposed in anti-scarring therapy to increase the safety and effectiveness and to reduce toxicity.

**Testing the Hypothesis:**

A chitosan-based polymeric predrug of MMC was synthesized and its cytotoxicity was proved to be low. In addition, we propose hyaluronic acid film as a container to release LDL-MMC-chitosan nanoparticles gradually at subconjunctival filtering site after glaucoma filtration surgery to eliminate the LDL-MMC-chitosan nanoparticles.

**Implications of the Hypothesis and discussion:**

This strategy can be applicable to anti-scarring therapy during excessive conjunctival wound healing. This hypothesis integrates advantages of the targeting drug delivery and antifibrotic agents, such as high efficiency, convenience, and lower the toxicity.

## Background

The most common cause of glaucoma filtration surgery failure is excessive scar formation at the surgical site[1,2]. The activation of HTFs is believed to be responsible for this problem[3,4], and local use of antiproliferative agents, such as mitomycin C (MMC) and 5-fluorouracil (5-FU), has improved the surgical success by preventing HTFs proliferation and scar formation. The traditional administration of these agents maintain the patency of the new filtration pathway. However, filtering blebs treated with these agents can still fail through scarring and because of their nonspecific mechanisms of action[5,6], these agents can cause widespread cell death and apoptosis. Thus, the traditional administration of these agents topically can result in a variety of complications, including blebitis, endophthalmitis, corneal epithelial defects, and hypotony with accompanying vision loss[7,8]. It is clinically well known that chronic tissue effects of antifibrotics (ie, to the conjunctiva) can occur for many years after a single topical application. Some authors argued that the toxicity of MMC to the ciliary epithelium might be an important contributor to its IOP lowering effect[9]. Because the antifibrotic agents are relatively nonspecific and react with cells in every phase in the cell cycle, they cause cytotoxicity by lipid peroxidation and DNA and protein damages[10].

These problems have stimulated the search for alternative modes of drug delivery and new agents to minimize the ocular complications[11]. Numerous implants that release MMC continuously have been investigated to avoid the naturally occurring scarring but cannot localize the side effects of the agent[12-14], for they cannot lower the toxicity to non-proliferating cells. Recently, studies have shown that constant nanoparticle drug delivery to targeted tissues and cells may offer a greater therapeutic effect than traditional dosing methods[15,16]. The results of other studies suggested that photosensitizer accumulates mainly inside the activated HTFs via the overexpressed LDLr to absorbs light and generates cytotoxic reactive oxygen species leading to cellular damage[17]. This result is confirmed *in vivo *by an investigation, using the photodynamic therapy (PDT) for anti-scarring after glaucoma filtering surgery [18]. Perhaps the most successful approval of PDT is with photosensitizer for injection (Visudyne) to treat age-related macular degeneration (AMD).

LDLr is a single-chain transmembrane glycoprotein that specifically mediates binding and endocytosis of LDL. Receptor-mediated endocytosis is a highly specific, high capacity process that can absorb a large amount of LDL into the cell within a relatively short time. Once internalized, LDLr dissociates from LDL and is recycled back to the cell surface where it is available to interact with many more LDL over its lifetime[19]. Then LDL are retained and accumulated in the sorting endosome. Therefore, any agent that can be attached to LDL can also be internalized and accumulate within LDLr expressing cells. In fact, the expression of the LDL receptors on the cell surface is regulated by the need of the cell for cholesterol[20]. It is known that rapidly proliferating tissues have a high demand for cholesterol for cell membrane synthesis. Increases of 3- to 100-fold in expression of LDLr over corresponding non-malignant tissues have been reported in acute myelogenous leukemia, colon cancer, adrenal adenoma, lung carcinoma, breast cancer, and prostate cancer[19,21].

Our previous study have shown that the LDLr are overexpressed in the activated HTFs[22]. As we showed, the LDLr protein in activated HTFs is 8-fold higher than that of normal HTFs, and the fluorescence of Dio-labeled LDL particles uptaked by activated HTFs is 3.73-fold higher than that of the normal HTFs. Therefore, we propose that the LDLr is a potential molecular target for the selective delivery of anti-scarring therapy during excessive conjunctival wound healing.

## Presentation of hypothesis

We propose that MMC encapsulated into LDL-chitosan nanoparticles (LDL-MMC-chitosan nanoparticles) will be an effective way to deliver MMC specifically to HTFs. The MMC-chitosan nanoparticles will be made as reports[23,24]. The nanocontainers are synthesized by well biodegradable, biocompatible, nonimmunogenic chitosan. Chitosan and its derivatives draw attention as a drug delivery vehicle. Especially, ordinary chitosan can be dissolved in acidic water but not in alkaline, that means the nanocontainers would degrade in the sorting endosome and release the MMC. Chitosan can easily react with many kinds of agents due to having -NH_2 _and -COOH groups in its structure, which is valuable for the drug carrier to readily prepare its conjugates with various drugs to avoid vexatious complications. That is possible to deal with the application of chitosan as a carrier for water-insoluble and water-soluble drug conjugates in anti-scarring therapy. Izume summarized the toxicity of chitosan including a skin sensitization study, temporal skin irritation study, ophthalmic sensitization test, mutagenicity test and patch test for humans; every study and test showed low toxicity of chitosan, which can function well as a drug carrier due to long systemic retention, low toxicity and accumulation in the target tissue[24-26].

In addition, firstly, nanoparticles increase uptake into activated HTFs that overexpressed LDLr, and steady release MMC. Secondly, chitosan nanoparticles confine and protect the enclosed MMC which may be hydrophilic until they bind to the outer membrane of the targeted HTFs, and lower effective dose of MMC. Thirdly, chitosan nanoparticles have the capability of keeping stability in human body. And finally, the scale dimensions of nanoparticles are between 1 and 100 nm, which favor endocytosis via LDLr.

The LDL particle is a naturally occurring nanostructure typically with a diameter of 22 nm. It contains a lipid core of some 1500 esterified cholesterol molecules and triglycerides. A shell of phospholipids and unesterified cholesterol surrounds this highly hydrophobic core. The shell also contains a single copy of apoB-100, which is recognized by the LDLr[25]. As reported that there are three ways to interact with LDL[19]: (1) via attachment to the apoB-100 protein on the surface of LDL, (2) via intercalation into the phospholipid monolayer of LDL, and (3) via substitution of the agent in the lipid core of LDL. Therefore, we can combine LDL and MMC-chitosan nanoparticle by one of the three strategies above.

## Testing the hypothesis

To test this hypothesis, a chitosan-based polymeric predrug of MMC was synthesized and the drug release rates were controlled by the aldehyde degree of chitosan and the mass ratios of periodate-oxidized chitosan (CS-CHO) to MMC (*m*_CS-CHO_/*m*_MMC_). When the *m*_CS-CHO_/*m*_MMC _was 5/1, 10/1 and 25/1, the initial release amount of MMC was 65%, 50% and 45%. There was an obvious initial release within the initial 8 h, and the concentration of MMC in dialysis medium remained unchanged during the following 60 h. Furthermore, the cytotoxicity study on chitosan-based polymeric predrug encapsulated fibroblast indicated that the maximum non-toxic concentration of CS-CHO was 8.3, 42.3 and 54.7 mg/ml in the 24, 48 and 72 h[26]. Therefore, the concentration of CS-CHO in the practical application should be lower than the corresponding concentration. *In vitro*, chitosan-based polymeric predrug of MMC was a low cytotoxic controlled delivery system.

**Figure 1:**
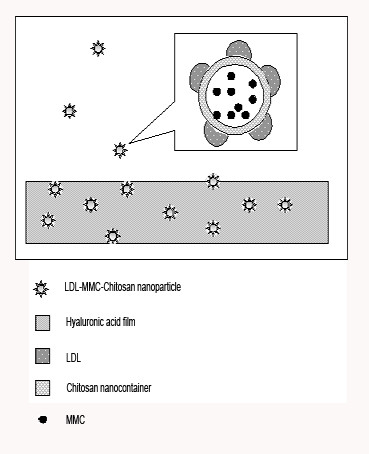
**LDL-MMC-chitosan nanoparticles release from hyaluronic acid film**. The hyaluronic acid film is implanted into subconjunctival space at the filtering site, with conjunctival wound is sutured, and LDL-MMC-chitosan nanoparticles release constantly from the hyaluronic acid film. LDL-MMC-chitosan nanoparticles is made by chitosan nanocontainer encapsulating MMC, and then combined with LDL.

In addition, we plan to insert LDL-MMC-chitosan nanoparticles into hyaluronic acid film (80 μm thickness) (Figure 1), which has no toxicity[27]. In glaucoma filtration surgery, the conjunctival wound is sutured with subconjunctival implantation of the hyaluronic acid film at the filtering site. In terms of physical characteristics, hyaluronic acid film changes from solid form into gel form within 24 to 48 hours in the tissue and stays within the tissue for about 7 to 14 days. However, it has been generally reported that fibroblasts increase the most at 4 to 7 days postoperatively. Furthermore, this film material functions as a barrier to contact between separated tissues by which the postoperative formation of adhesions is reduced and delayed, and no subsequent removal procedure is required. With the gradual degradation of hyaluronic acid film in the body, the LDL-MMC-chitosan nanoparticles will be released into the subconjunctival space, and endocytosed mainly by activated HTFs at wound site.

We will observe the validity of creating a long-term effective filtering bleb and decrease of the ocular complications, compared with the control. The encapsulation allows constant release rather than a burst of drugs so that a high therapeutic efficiency can be achieved without side effects. The LDL-MMC-chitosan nanoparticles drug delivery system is proposed based on the LDL receptor endocytosis pathway. The receptor-mediated uptake mechanism is an interesting aspect of the LDLr system. Receptor-mediated endocytosis is a highly specific, high capacity process that can absorb a large amount of LDL-MMC-chitosan nanoparticles into the activated HTFs within a relatively short time. Once internalized, LDLr dissociates from LDL-MMC-chitosan nanoparticles and is recycled back to the cell surface where it is available to interact with many more LDL-MMC-chitosan nanoparticles over its lifetime. With a recycle time of approximately 10 min and a lifetime of about 24 h, one can assume that each receptor mediates the transport of about 144 LDL-MMC-chitosan nanoparticles into the activated HTFs per day. Assuming about 1,000 receptors per cell, this provides an extremely efficient system for delivering its MMC. Therefore, LDL-MMC-chitosan nanoparticles accumulate mainly within the activated HTFs instead of bring nonspecific toxicity to both proliferating and non-proliferating cells.

## Implications of the Hypothesis and discussion

In this article, we propose LDL-MMC-nanoparticles as a consistent drug delivery system that specifically bind to LDLr mainly in activated HTFs. This may achieve a long circulation time, low immunogenicity, good biocompatibility, highly selective targeting, lower drug dose, reduced toxicity to normal cells, and increased safety and effectiveness of anti-scarring therapy during excessive conjunctival wound healing.

## Competing interests

The authors declare that they have no competing interests.

## Authors' contributions

TS conceived the hypothesis and drafted the manuscript; XL carried out the study of MMC-chitosan nanoparticles synthesis; JG revised the manuscript critically and gave final approval of the version to be published. All authors read and approved the final manuscript.
